# Effects of Biochar and PGPR Application on the Physicochemical Properties and Humus Components of Soil Used for Planting Fruit Mulberry Seedlings Under Salt Stress

**DOI:** 10.3390/biology14101441

**Published:** 2025-10-18

**Authors:** Dawei Jing, Fangchun Liu, Binghua Liu, Lin Peng, Mingjie Sun, Hailin Ma, Zhenyu Du

**Affiliations:** 1 College of Ecology, Resources and Environment, Dezhou University, Dezhou 253023, China; 2 Institute of Resource and Environment, Shandong Academy of Forestry, Jinan 250014, China; 3 Shandong Academy of Forestry, Jinan 250014, China

**Keywords:** *Morus atropurpurea* ‘Zibingmoyu’ seedling, biochar, plant growth-promoting rhizobacteria, physicochemical properties, humus components

## Abstract

**Simple Summary:**

Saline–alkali land typically exhibits poor soil properties, and improving its soil quality and productivity is a critical scientific challenge. In this study, we investigated the effects of biochar and *Bacillus fexus* application on the physicochemical properties, humus components, and their stability in soil when planting fruit mulberry seedlings under salt stress. Our results showed that the simultaneous addition of biochar and *Bacillus fexus* under salt stress significantly ameliorated soil properties: it increased soil total porosity, non-capillary porosity, the contents of organic matter, humic acid, and humin, and the ratios of humic acid to fulvic acid and humic acid to humin. Conversely, it significantly reduced soil bulk density and humin to humus-extractable ratio. These findings suggest that the combined application of biochar and *Bacillus fexus* under salt stress can effectively optimize soil physicochemical properties and enhance soil humus component content and humus stability. This approach is of great significance for improving the soil quality of saline–alkali land and boosting the productivity of fruit mulberry.

**Abstract:**

Biochar can act as a carrier and a soil carbon source for rapid colonization by plant growth-promoting rhizobacteria (PGPR). However, the effects of a combined application of biochar and PGPR on soil physicochemical properties, humus components, and their stability in the rhizosphere around fruit mulberry seedlings remain unclear. A pot experiment using 1-year-old fruit mulberry seedlings with five treatments (control (CK), salt stress (SS), salt stress + *Bacillus fexus* (SS+P), salt stress + biochar (SS+B), and salt stress + *B. fexus* + biochar (SS+P+B)) was conducted to analyze the variations in soil physicochemical properties and humic acid (HA), fulvic acid (FA), and humin (HM) contents in the soil when planting fruit mulberry seedlings. The results indicated that the SS treatment significantly reduced total soil porosity, non-capillary porosity, water stable macro-aggregates content, available potassium content, and pH value compared to CK, but increased the soil bulk density, capillary porosity, and available phosphorus content. The SS+P+B treatment significantly increased soil total porosity, non-capillary porosity, pH value, electrical conductivity, the water stable macro-aggregates, organic matter, HA and HM contents, the HA/FA and HA/HE (humus-extractable) ratios, and the activities of catalase and urease. It significantly increased the water stable macro-aggregates and the HA/HE ratio by 27.83% and 25.00%, respectively. However, it significantly decreased soil bulk density and capillary porosity by 9.93% and 20.64%, respectively, compared to the SS treatment. The results suggest that the simultaneous addition of biochar and *B. fexus* under salt-stress conditions improves the soil physicochemical properties and increases the humus components content and stability, which is of great significance for improving the soil quality of saline–alkali land and enhancing the productivity of fruit mulberry.

## 1. Introduction

Soil salinization is a globally important environmental issue. At present, the global soil area affected by salinization is as high as 9.6 × 10^8^ hm^2^. Furthermore, the total area covered by various types of saline–alkali land in China is approximately 3.6 × 10^7^ hm^2^; it accounts for 4.88% of all available area and is increasing year by year [1]. Fruit trees are important economic tree species. The promotion of fruit tree production in saline–alkali land will increase the use of the underused saline–alkali land and could achieve considerable economic benefits. However, saline–alkali land usually has poor soil characteristics, such as a heavy and compact texture, poor air and water permeability, and a low organic matter content. It also loses considerable amounts of humus due to leaching. These characteristics have restricted the sustainable development of the fruit tree industry. Therefore, exploring approaches to synergistically improve soil physicochemical properties and enhance soil quality and land productivity in saline–alkali regions has become a critical scientific issue that needs to be addressed.

Plant growth-promoting rhizobacteria (PGPR) is the general term for beneficial bacteria that inhabit the rhizosphere around plants and promote plant growth or antagonize pathogenic bacteria [2,3]. The PGPR are involved in soil functions such as nitrogen fixation, phosphorus solubilization, potassium mobilization, antibiotic secretion, and hormone synthesis [4]. They can also improve soil aggregate structure and chemical properties and increase the number of non-capillary pores in the soil, thereby enhancing soil water retention and aeration, which leads to a better environment for plant growth [5,6]. In recent years, PGPR have been increasingly applied in agricultural and forestry production and are expected to partially replace chemical fertilizers and pesticides in the future. El-Amriti et al. [7] found that the selected bacteria *Alcaligenes *sp. E1 and E2 significantly improved the overall growth attributes of maize under salt stress (150 mM NaCl) in a greenhouse experiment. Ullah et al. [8] reported that using PGPR induced salt tolerance in sunflowers. However, in some environments, exogenous PGPR entering the soil may not survive due to sudden environmental changes and competition from indigenous strains [9]. Therefore, an excellent carrier is needed to ensure that the PGPR survive in new environments. A suitable carrier should have good air permeability and provide basic nutrients and protection against predation by soil animals. These characteristics will improve PGPR inoculation success rates [10].

Biochar is a carbon-rich material produced by the high-temperature pyrolysis of biomass, such as agricultural and forestry wastes, under anaerobic or hypoxic conditions [11]. It can be made from low-cost and easily available raw materials and has a highly microporous structure, abundant surface functional groups, and a large specific surface area [12,13]. It has been widely studied and applied in agricultural and forestry production. Wang et al. [14] found that biochar addition reduced the soil bulk density and increased the organic matter, alkaline-hydrolyzable nitrogen, available phosphorus, and available potassium contents in rhizosphere soils under saline–alkali stress; Eghlima et al. [15] reported that biochar increased the porosity, water-holding capacity, and enzyme activity of soils under salinity stress; and Kim et al. [16] found that biochar applications increased the soil organic carbon content and the percentage of water stable aggregates. Furthermore, biochar has a highly aromatic structure that is similar to soil humus [17], which accounts for more than 50% of soil organic matter [18]. Humus is the main reservoir for soil organic carbon and plays a crucial role in promoting soil structure formation and nutrient accumulation [19]. It can be classified into three components based on its solubility in acidic or alkaline solutions: humic acid (HA), fulvic acid (FA), and humin (HM).

The application of biochar to soils can promote the humification of soil organic matter and the formation of humus [20,21]. Song et al. [22] found that the FA and HM contents in cadmium-contaminated soil increased with the increase in biochar addition, whereas the HA content decreased. Furthermore, Amoakwah et al. [23] reported that the application of corn cob biochar to a tropical sandy loam increased the HA and FA concentrations and led to increased stratification of total organic carbon, with a stronger effect on HA than on FA. Other studies have shown that biochar application can induce significant changes in the soil microbial community, altering the composition of the soil microorganisms and the abundance of related species [24]. These results suggest that biochar promotes the proliferation of soil microorganisms and relevant changes may drive nutrient cycling, thereby directly or indirectly affecting plant growth [25]. However, little research is available on the combined application of biochar and microorganisms.

Mulberry (*Morus alba* L.) has significant nutritional, medicinal, and economic value [26]. Mulberry is highly adaptable and plays an important role in soil and water conservation, saline–alkali soil improvement, and urban landscaping [27]. Fruit mulberry is a type of mulberry cultivated for fruit production, which is nutrient-rich and abundant in bioactive components that have healthcare functions [28]. Previous research on mulberry under salt stress mainly focused on physiological responses and molecular-level mechanisms [27,29,30]. There have been relatively few studies on soil humus components, especially the effects of combined applications of biochar and PGPR on the physicochemical properties, humus components, and their stability in soil for planting fruit mulberry.

Thus, we used 1-year-old fruit mulberry seedlings as test materials in a pot experiment to investigate the effects of biochar and PGPR applications on the soil’s physicochemical properties, humus components, and stability, as well as the enzyme activity around fruit mulberry seedlings under salt stress. The objectives were to clarify the effect of a combined application of biochar and PGPR on soil humus under salt stress, reveal the relationship between soil physicochemical properties and humus fractions, and provide a theoretical basis and technical support for the application of biochar and PGPR to improve the fertility and quality of saline–alkali soils.

## 2. Materials and Methods

### 2.1. Bacterial Selection and Identification

A bacterial strain with known positive effects on plant growth was used in the present experiment and preserved in the China General Microbiological Culture Collection Center (CGMCC No. 31832, Beijing, China) on 4 September 2024. The bacterial strain E1 was isolated and screened from the rhizosphere soil of a black locust forest in Dongying City, Shandong Province, China, using the serial dilution method and bioassay, which possessed functions such as organophosphorus degradation, IAA production, and exopolysaccharides production.

Based on the 16S rRNA sequencing data and the comparison of its biochemical characteristics (Table 1) to those described in Bergey’s Manual of Determinative Bacteriology [31], the bacterial strain was indeed identified as *Bacillus fexus*. The 16S RNA gene sequence of this strain is provided in Supplementary Material File S1.

### 2.2. Basic Soil and Plant Materials

The trial from March to June 2025 was carried out in the plant nursery of Shandong Academy of Forestry, Taian (36°23′ N, 117°27′ E), Shandong Province, China.

The experimental soil was loam, and the basic physical and chemical properties were as follows: alkali-hydrolyzable N: 39.2 mg/kg, available P: 9.07 mg/kg, available K: 96.52 mg/kg, organic matter: 18.51 g/kg, pH 6.83, electrical conductivity (EC): 181.5 μS/cm.

The seedlings used in the experiment were 1-year-old fruit mulberry seedlings (*Morus atropurpurea* ‘Zibingmoyu’).

The peanut shell biochar used was purchased in powder form from Henan Lvtanyuan Biotechnology Co., Ltd. (Luoyang, China), and its basic physical and chemical properties are shown in Table 2.

### 2.3. Experimental Design

The experiment was conducted using plastic pots purchased from a local market. The pots were 28 cm high and had an outer diameter of 32.4 cm at the top and an outer diameter of 21.5 cm at the bottom. On 28 March 2025, approximately 11.36 kg of soil was placed in each pot, and a 1-year-old fruit mulberry seedling was transplanted into each pot. After transplantation, all the seedlings were uniformly pruned to a height of 40 cm. There were five treatments with 10 pots in each treatment: (1) CK (control, no salt stress); (2) SS (salt stress); (3) SS+P (salt stress + PGPR); (4) SS+B (salt stress + biochar); and (5) SS+P+B (salt stress + PGPR + biochar). The biochar in the treatments of SS+B and SS+P+B was added at 5% of soil weight and was thoroughly mixed in with the soil. The NaCl dose was 0.3% of the soil weight in the treatments of SS, SS+P, SS+B, and SS+P+B, and the total amount of NaCl applied to each pot was divided into five equal applications. The NaCl solution was applied once every other day, starting on 9 May 2025, and there were five applications in total. For each application, one portion of NaCl was dissolved in 150 mL solution, which was then slowly and uniformly poured around the root zone of the mulberry seedling. The CK group received 150 mL of tap water. The SS+P and SS+P+B treatments were inoculated with the PGPR three times on 20 May 2025, 21 May 2025, and 22 May 2025, respectively. For each inoculation, 20 mL of bacterial solution (*Bacillus fexus* E1) was first diluted to 300 mL and then the diluted solution was evenly and slowly poured onto the root zone of the mulberry seedling. All treatments received small, frequent applications of water to prevent salt leaching. The experiment was terminated when severe leaf wilting was observed (25 June 2025).

### 2.4. Data Collection and Determinations

After the salt stress experiment was terminated on 25 June 2025, an undisturbed topsoil sample was collected from the pot using a 100 cm^3^ cutting ring. This sample was then used to determine the soil bulk density and field water-holding capacity. Approximately 2 kg of large undisturbed soil clods were also collected to determine the soil water-stable aggregate content (>0.25 mm), and the rhizosphere soil was collected according to Wang and Zabowski [32] to determine the soil chemical properties, humus components, and enzyme activities.

To determine soil pH (water: soil 2.5:1) and EC (water: soil 5:1), a pH meter and a conductivity meter were used, respectively. Alkali-hydrolyzed N in soil was determined by the alkali-hydrolyzed diffusion method, available P by the NaHCO_3_ extraction–molybdenum antimony anti-colorimetry method, and available K by the NH_4_OAc extraction-flame photometry method [33].

The bulk density, field water-holding capacity, water stable macro-aggregates, soil organic matter (SOM), cation exchange capacity (CEC), humus components, and soil enzyme activities were determined by the Shandong Artisan Testing Co., Ltd. (Weifang, China) using the following methods.

Soil bulk density and field water-holding capacity were determined using the cutting ring method and the corresponding calculation equations were as follows:

Total porosity (%) = (1 − bulk density/2.65) × 100(1)

Capillary porosity (%) = field water-holding capacity × bulk density × 10^−1^ (2)

Non-capillary porosity (%) = Total porosity − capillary porosity.(3)

SOM, CEC, and water stable macro-aggregate (>0.25 mm) were measured using the potassium dichromate volumetric method, the ammonium acetate exchange method, and the wet sieve method, respectively. Humus components (HA, FA, and HM) were determined by the sodium pyrophosphate–sodium hydroxide extraction combined with the potassium dichromate oxidation volumetric method.

Catalase was determined by the KMnO_4_ titration method, urease by the sodium phenolate–sodium hypochlorite colorimetric method, sucrase by the 3,5-dinitrosalicylic acid colorimetric method, and neutral phosphatase by the disodium phenyl phosphate colorimetric method.

### 2.5. Statistical Analyses

The experiments were performed using a completely randomized design. The data were compared statistically among the five different treatments using analysis of variance (ANOVA) in SPSS (version 25.0) and least significant difference (LSD) as post hoc tests to distinguish the treatment groups. Differences were statistically significant at (*p* < 0.05). Principal component analysis scatter plots and correlation analysis were conducted using OriginPro 2025 software. All data are expressed as means ± standard deviation (SD) of three replicates.

## 3. Results

### 3.1. Soil Physical Properties

The physical properties of the soil used for planting the fruit mulberry seedlings differed among the treatments (Table 3). The field water-holding capacity variation range among the treatments was 185.00–209.00 g/kg, but there were no significant differences among the treatments. The bulk density and capillary porosity were greatest in the SS treatment and had significantly increased by 6.02% compared to CK. However, no significant difference was observed in CP between CK and SS treatments. Bulk density and capillary porosity were lowest in SS+P+B and had decreased by 9.93% and 20.64%, respectively, compared to the SS treatment. Furthermore, the SS+P and SS+B treatments also had lower bulk densities and capillary porosities than the SS treatment. Table 3 also showed that the total porosity and non-capillary porosity were lowest in SS-treated soil, where they were significantly lower than those for the CK, SS+B, and SS+P+B treatments. However, total porosity and non-capillary porosity in the SS+P+B treatment were significantly greater than in the other treatments. In particular, non-capillary porosity in the SS+P+B treatment was significantly greater than in the CK, SS, SS+P, and SS+B treatments, by 20.82%, 67.72%, 39.64%, and 17.42%, respectively. The SS treatment had the lowest water stable macro-aggregate content, which had significantly decreased by 7.99% compared to CK. In contrast, the SS+P+B treatment had the largest water stable macro-aggregate content, followed by the SS+B treatment. There were no significant differences between the SS+P+B and SS+B treatments, but they had significantly higher water stable macro-aggregate contents than the SS treatment by 27.83% and 22.19%, respectively.

The above results suggested that salt stress increased the bulk density and capillary porosity in the soil used for planting fruit mulberry seedlings, but reduced the total porosity, non-capillary porosity, and the water stable macro-aggregate content. However, when the soil was under salt stress, the addition of PGPR or biochar reduced the bulk density and capillary porosity in the soil by varying degrees and increased the total porosity, non-capillary porosity, and the water stable macro-aggregate contents compared to SS. The effect was most obvious when PGPR and biochar were added into soil simultaneously.

### 3.2. Basic Chemical Properties in Rhizosphere Soil

The soil pH in the SS treatment was significantly lower than that of CK (Table 4). In contrast, the pH values in the SS+B and SS+P+B treatments significantly increased by 0.28 and 0.39, respectively, compared to the SS treatment. The EC values for the four salt-stress treatments significantly increased compared to CK. The SS+P+B treatment had the greatest EC value, followed by SS+B, SS, and SS+P. The alkali-hydrolyzed N contents of the treatments ranged from 28.00 to 32.20 mg/kg and the contents in the four salt-stress treatments all decreased compared to CK, but the differences among these treatments were not significant. Table 4 also showed that the available P content in the SS treatment was slightly higher than that in CK, but the difference was not significant. However, the available P contents of the SS+B and SS+P+B treatments were significantly lower than that of the SS treatment. The available K content in the SS treatment was significantly lower than that of CK by 9.64%, whereas the available K content in SS+B was significantly higher than that of SS by 22.87% and the available K content in SS+P+B was significantly lower than that of SS. Additionally, both SOM content and CEC were the greatest in SS+P+B treatment. There was no significant difference in SOM content between the SS+P+B and SS+B treatments. However, both were evidently higher than those for the other three treatments, with significant increases of 41.43% and 34.63% compared to the SS treatment, respectively. The CEC for SS+P+B was significantly higher than that of CK, but there were no significant differences between SS+P+B and the other three treatments.

The results suggest that salt stress altered the chemical properties of fruit mulberry seedling rhizosphere soil, whereas the addition of PGPR or biochar under salt stress mitigated these effects to varying degrees.

### 3.3. Humus Components in Rhizosphere Soil

**Figure 1:**
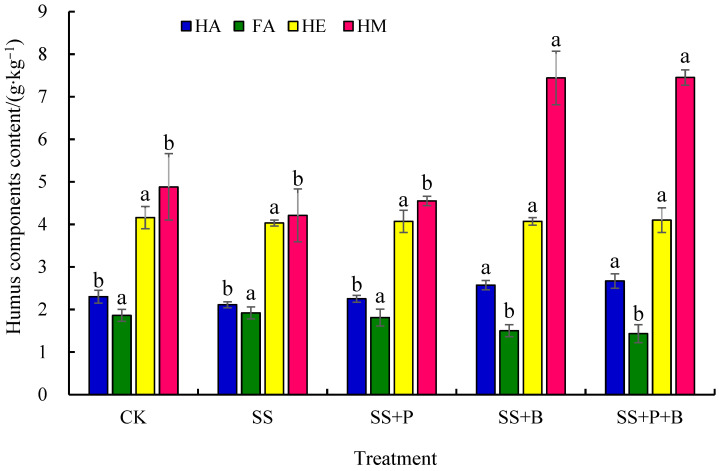
Effects of different treatments on the content of humus components in fruit mulberry seedling rhizosphere soil. Bars are means, and error bars are standard deviations (*n* = 3). CK: control; SS: salt stress; SS+P: salt stress + PGPR; SS+B: salt stress + biochar; SS+P+B: salt stress + PGPR + biochar; HA: humic acid; FA: fulvic acid; HE: humus-extractable; HM: humin. Different letters indicate significant differences among treatments at *p* < 0.05 by LSD.

The HA and HM contents were greatest in the SS+P+B treatment, followed by the SS+B treatment (Figure 1). There were no significant differences in the HA and HM contents between the SS+P+B and SS+B treatments, but both were significantly higher than those for the other three treatments. Specifically, the HA contents in the SS+P+B and SS+B treatments significantly increased by 26.54% and 21.80%, and the HM contents significantly increased by 76.96% and 76.72%, respectively, compared to the SS treatment. The SS treatment also had the highest FA content, followed by the CK and SS+P treatments. There were no significant differences in FA content among these three treatments, but their FA contents were all significantly higher than those in the SS+B and SS+P+B treatments. The FA content was significantly lower in the SS+P+B treatment by 23.12% and 25.52% compared to the CK and SS treatments, respectively. Furthermore, the HE contents in the four salt-stress treatments all decreased to a certain extent compared to CK, but the differences among the four treatments were not significant.

The above analysis indicated that under salt-stress conditions, the addition of biochar or the simultaneous addition of PGPR and biochar could increase the HA and HM contents in fruit mulberry seedling rhizosphere soil, whereas the FA content would be reduced.

### 3.4. Humus Stability in Rhizosphere Soil

**Figure 2:**
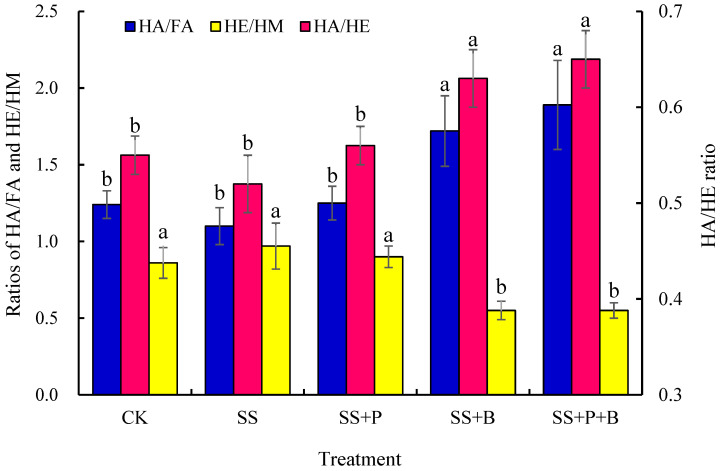
Effects of different treatments on the stability of humus in fruit mulberry seedling rhizosphere soil. Bars are means, and error bars are standard deviations (*n* = 3). CK: control; SS: salt stress; SS+P: salt stress + PGPR; SS+B: salt stress + biochar; SS+P+B: salt stress + PGPR + biochar; HA: humic acid; FA: fulvic acid; HE: humus-extractable; HM: humin. Different letters indicate significant differences among treatments at *p* < 0.05 by LSD.

The larger the HA/FA and HA/HE ratios, the more stable the soil humus becomes, whereas the larger the HE/HM ratio, the stronger the soil humus activity [34]. Each treatment had a certain influence on humus stability in the rhizosphere soil (Figure 2). The HA/FA and HA/HE ratios were greatest in the SS+P+B treatment, followed by the SS+B treatment. There were no significant differences in these two ratios between the SS+P+B and SS+B treatments, but both were significantly higher than those in the other three treatments. The HA/FA ratio significantly increased by 71.82% and 56.36%, and HA/HE significantly increased by 25.00% and 21.15% in the SS+P+B and SS+B treatments, respectively, compared to the SS treatment. The HA/FA and HA/HE ratios were lowest in the SS treatment, but there were no significant differences among the SS, CK, and SS+P treatments. The HE/HM ratio was greatest in the SS treatment, but it was not significantly different from the CK and SS+P treatments. However, it was significantly higher than those in the SS+B and SS+P+B treatments. The HE/HM ratio in the SS+P+B treatment significantly decreased by 43.30% compared to the SS treatment, which suggests that under salt stress, the addition of biochar alone or the combined addition of PGPR and biochar could improve humus stability and reduce humus activity in fruit mulberry seedling rhizosphere soil.

### 3.5. Rhizosphere Soil Enzyme Activity

The catalase and urease activities in the rhizosphere soil differed among the treatments (Table 5) and were greatest in the SS+P+B treatment. In particular, the catalase activity significantly increased by 16.67% and 15.19% and the urease activity significantly increased by 175.00% and 160.00% compared to the CK and SS, respectively. The catalase activities in the SS+P and SS+B treatments were not significantly different from SS+P+B, and the urease activity in the SS+B treatment was not significantly different from that of SS+P+B, but was significantly greater than in CK, SS, and SS+P. The variation range for sucrase activity was 29.53–42.20 mg/g·24 h and was lower in the SS treatment than in CK. However, the sucrase activity was increased in the treatments of SS+P, SS+B, and SS+P+B compared to the SS treatment, but the differences among the five treatments were not significant. Furthermore, there were no significant differences in neutral phosphatase activity among the treatments.

The results suggested that under salt-stress conditions, the simultaneous addition of PGPR and biochar could increase the catalase, urease, and sucrase activities to varying degrees, which would improve the microenvironment of fruit mulberry seedling rhizosphere soil.

### 3.6. Relationship Between Soil Physicochemical Properties and Humus Components

**Figure 3:**
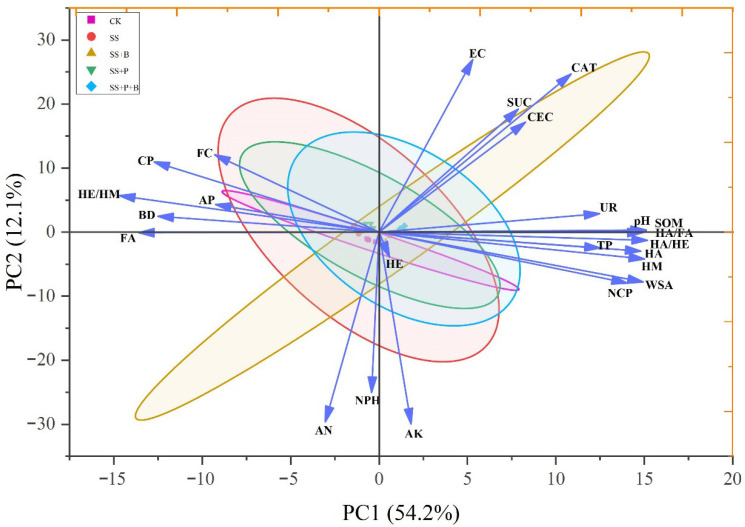
Scatter plot of principal component analysis. FC: field water-holding capacity; TP: total porosity; CP: capillary porosity; NCP: non-capillary porosity; WSA: water stable macro-aggregate; EC: electrical conductivity; AN: alkali-hydrolyzed nitrogen; AP: available phosphorus; AK: available potassium; SOM: soil organic matter; CEC: cation exchange capacity; HA: humic acid; FA: fulvic acid; HE: humus-extractable; HM: humin; CAT: catalase; UR: urease; NPH: neutral phosphatase; SUC: sucrase.

**Figure 4:**
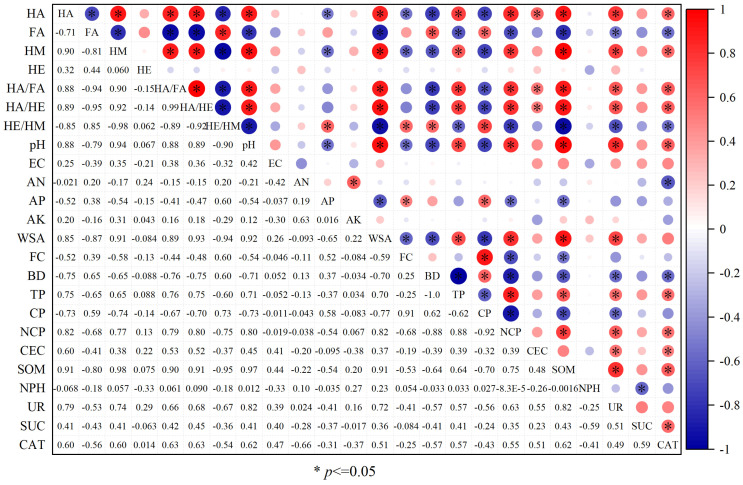
Correlation analysis of humus components with soil properties and enzyme activities. FC: field water-holding capacity; TP: total porosity; CP: capillary porosity; NCP: non-capillary porosity; WSA: water stable macro-aggregate; EC: electrical conductivity; AN: alkali-hydrolyzed nitrogen; AP: available phosphorus; AK: available potassium; SOM: soil organic matter; CEC: cation exchange capacity; HA: humic acid; FA: fulvic acid; HE: humus-extractable; HM: humin; CAT: catalase; UR: urease; NPH: neutral phosphatase; SUC: sucrase.

Principal component analysis of soil physicochemical properties, enzyme activity, and humus components indicated that the contribution rates of the two principal components were 54.2% and 12.1%, respectively (Figure 3). Pearson correlation analysis (Figure 4) showed that HA was significantly positively correlated with HM, HA/FA, HA/HE, pH, WSA, TP, NCP, CEC, SOM, UR, and CAT. It was significantly negatively correlated with FA, HE/HM, AP, FC, BD and CP. FA was significantly positively correlated with HE/HM, BD, and CP, and significantly negatively correlated with HM, HA/FA, HA/HE, pH, WSA, TP, NCP, SOM, UR, and CAT. HM was significantly positively correlated with HA/FA, HA/HE, pH, WSA, TP, NCP, SOM, UR, and CAT, and significantly negatively correlated with HE/HM, AP, FC, BD, and CP. HA/FA was significantly positively correlated with HA/HE, pH, WSA, TP, NCP, CEC, SOM, UR, and CAT, and significantly negatively correlated with HE/HM, BD, and CP. HA/HE was significantly positively correlated with pH, WSA, TP, NCP, CEC, SOM, UR, and CAT, and significantly negatively correlated with HE/HM, BD, and CP. HE/HM was significantly positively correlated with AP, FC, BD, and CP, and significantly negatively correlated with pH, WSA, TP, NCP, SOM, UR, and CAT. The above analysis showed that pH, WSA, TP, NCP, SOM, UR, and CAT had a significant correlation with the content of humus components, and pH, WSA, TP, NCP, CEC, SOM, UR, and CAT had a significant positive correlation with the stability of soil humus. Meanwhile, AP, FC, BD, and CP were significantly positively correlated with the activity of soil humus.

## 4. Discussion

### 4.1. Soil Physical and Chemical Properties

Soil bulk density reflects the compactness and water-holding capacity of the soil, whereas soil porosity reflects the aeration and water permeability of the soil. They are closely related and jointly affect soil moisture, nutrients, aeration, and temperature [35]. Soil aggregates with particle sizes > 0.25 mm are the main storage reservoir for soil nutrients and are an important microenvironment for microbial activities [36]. In this study, the bulk density of the soil used for planting the fruit mulberry seedlings significantly increased under salt stress, and the corresponding capillary porosity and field water-holding capacity reached their maximum values, whereas the total porosity, non-capillary porosity, and water stable macro-aggregate significantly decreased. However, the addition of biochar under salt-stress conditions could significantly reduce soil bulk density and capillary porosity, and significantly increase total porosity, non-capillary porosity, and water stable macro-aggregate This might be attributed to the fact that biochar has a rich porous structure that provides a “skeleton” for soil particles to bond to. This would promote the aggregation of small particles into large aggregates, which would increase the non-capillary porosity and aeration of the soil and enhance total porosity. Furthermore, biochar not only improves the soil microenvironment, but also increases the SOM content, which would stimulate microbial activity [37]. Microorganism activities and their secretions can promote the formation of aggregates with particle sizes > 0.25 mm. This would increase the non-capillary porosity of the soil and decrease its bulk density. These results are similar to those reported by Liu et al. [38] for *Melissa officinalis* L. The results suggest that adding both biochar and PGPR simultaneously has significant synergistic effects. This might be due to the fact that biochar provides a favorable living environment for PGPR. This enables the PGPR to increase microbial richness and encourages microorganisms to generate organic metabolites. These metabolites enable microaggregates to aggregate into macroaggregates [39] and increase non-capillary porosity.

The results also showed that the pH value decreased and EC significantly increased in the rhizosphere soil under salt stress. However, after adding biochar to the soil under salt stress, the soil pH value significantly increased. The reason may be that biochar itself is alkaline and contains base cations, such as Ca^2+^ and Mg^2+^, which enter the soil and exchange with ions such as Al^3+^ and H^+^. This would increase base saturation and regulate the pH of acidic soils [40]. These results were generally consistent with the results reported by Mendes et al. [41]. Meanwhile, the cations in biochar are released and dissolved into the soil solution. However, the entire salt stress experiment was conducted under no-leaching conditions, which ultimately led to an increase in soil salt concentration. Cao et al. [42] found that the addition of biochar increased the soil pH value and EC, which was similar to the results from this study. However, Chen et al. [43] and Huang et al. [44] reported that biochar application significantly decreased soil EC under salt stress. This was not consistent with the results from this study. The reasons for this discrepancy may be related to factors such as the inherent properties of the biochar (feedstock, pyrolysis process, and post-treatment method), the dosage and duration of the biochar application, and whether leaching occurred during the experimental process.

In this experiment, the addition of biochar to soil under salt stress significantly increased the SOM levels. This was similar to the results reported by Huang et al. [44]. The simultaneous addition of biochar and PGPR resulted in the largest increase in SOM, which indicated that the combined application of biochar and PGPR had a synergistic effect. This might be due to the rich porous structure of biochar providing an ecological niche for soil colonization by PGPR [25]. This niche then enabled the PGPR to resist invasion by other, less favorable organisms [45]. Meanwhile, under salt-stress conditions, the addition of biochar can increase the available K content in the rhizosphere soil. This may be related to the fact that biochar itself has relatively high water-soluble potassium and exchangeable potassium contents [46]. However, the simultaneous addition of biochar and PGPR decreased the alkali-hydrolyzed N, available P, and available K contents in the rhizosphere soil. A possible reason for this might be that biochar not only acts as a colonization carrier for PGPR but also offers carbon sources for its rapid reproduction and promotes the growth and activity of microorganisms. These characteristics increase extracellular enzyme activities in the soil, which facilitates the absorption and utilization of soil nutrients such as nitrogen, phosphorus, and potassium by fruit mulberry seedlings. Furthermore, the addition of biochar and PGPR significantly increased the total porosity and non-capillary porosity of the soil, which would promote root growth and enhance the absorption and utilization of soil nutrients by fruit mulberry seedlings [47].

### 4.2. Humus Components and Stability in Rhizosphere Soil

Soil humus is the cementing material for aggregates, and its formation and transformation maintain the stability of aggregates [48]. The application of biochar can alter the soil humus component content, and the accumulation and decomposition of these components will further affect soil fertility [49]. In this study, the addition of biochar under salt-stress conditions significantly increased the HA content in the rhizosphere soil but significantly reduced the FA content. The reason for this might be that biochar contains aliphatic and oxidized carbon, which provide carbon and energy sources for microorganisms, improve their living environment, and enhance their decomposition ability, quantity, and activities [50]. Soil FA is the most unstable component in humus and is the first to be decomposed or polymerized by microorganisms to form HA. This leads to a significant increase in soil HA content after biochar application. Furthermore, FA has a smaller molecular weight than HA. The biochar preferentially adsorbs small-molecule organic substances in the soil, resulting in a decrease in FA content [34]. However, Wang et al. [51] reported that the C contents in HA and FA remained unchanged as the biochar application rate increased, and Wang et al. [52] showed that rice husk, tobacco stalk, and corn stalk biochar all increased the soil FA content. Ying et al. [53] found that rice straw significantly increased the extractable humus, HA, and FA contents in soil, whereas biochar only significantly affected HA and humic degree values. These studies suggest that changes in the application time and dosage of biochar can affect the content and quality of the soil nutrients [54] and may also be related to factors such as the inherent properties of the biochar and the experimental environment.

The results from this study suggested that applying biochar under salt-stress conditions significantly increased the HM content in the rhizosphere soil. This was possibly due to the low activity and relatively stable nature of HM, which gives it similar properties to biochar. Relevant studies reported that the C content in HM strongly increased as the biochar application rate increased [51]. This was consistent with the results from this experiment. Furthermore, ^13^C isotope tracer studies have also indicated that the biochar applied to the soil mainly became part of the HM component [55], which directly increased the HM content in the soil.

The humification degree and molecular complexity of soil humus can be measured using the HA/FA or HA/HE ratios, and the quality of the soil humification process can be determined from the HA/FA and HA/HE ratios [56]. In this study, the application of biochar under salt stress significantly increased the HA/FA and HA/HE ratios in the rhizosphere soil, but decreased the HE/HM ratio, which suggested that biochar application improved humification rates and humus stability in the rhizosphere soil around mulberry seedlings. The reason might be that after applying biochar, the HA condensation degree and aromaticity in the soil increased, the oxidation degree decreased, and the molecular structure became more complex [57]. This would increase the stability of the HA in the soil and decrease its decomposition into FA. This result may also be related to the fact that FA is more easily adsorbed by biochar.

### 4.3. Enzyme Activity in Rhizosphere Soil

Soil enzymes, such as catalase, urease, sucrase, and phosphatase, are mainly derived from secretions by microorganisms, animals, and plants and the decomposition and release of animal and plant residues. Wang et al. [51] and Hou et al. [58] reported that biochar application can enhance the catalase, urease, and sucrase activities in the soil. In this study, there were no significant changes in the catalase and urease activities compared to CK in the rhizosphere soil under salt stress without biochar. However, the addition of biochar under salt-stress conditions increased the catalase and urease activities to varying degrees, and the largest increase occurred when both biochar and PGPR were added simultaneously. Possible reasons for this might be that biochar improves soil structure by ameliorating the physical and chemical properties of the salt-stressed soil around the fruit mulberry seedlings and provides a carrier for soil enzymes [59], and secondly, in addition to providing some nutrients required for microorganism growth, its rich pore structure has a strong ability to retain water and nutrients, which would provide a relatively suitable habitat and serve as a metabolic substrate for microorganisms [60]. Moreover, the increased microorganism reproduction rate would increase soil enzyme activities. Thirdly, the adsorption of reaction substrates by biochar can provide more binding sites for soil enzymes, thereby increasing the enzymatic reaction rate.

Biochar application did not have a significant effect on neutral phosphatase activity in the rhizosphere soil. The reason for this might be that the surface functional groups on biochar are similar to phosphorus-containing organic matter and can bind to phosphatase through electrostatic interaction. This would exert a competitive inhibitory effect on phosphatase [61].

## 5. Conclusions

The results of this study indicate that the simultaneous addition of biochar and *Bacillus fexus* under salt stress significantly improved multiple soil properties: it increased soil total porosity, non-capillary porosity, the contents of water stable macro-aggregate, organic matter, humic acid, and humin, and the ratios of humic acid to fulvic acid and humic acid to humin, as well as catalase and urease activities. Conversely, it significantly reduced soil bulk density, capillary porosity, available phosphorus content, available potassium content, fulvic acid content, and humin to humus-extractable ratio. Therefore, the combined application of biochar and *Bacillus fexus* under salt stress can effectively optimize soil physicochemical properties and enhance soil humus component content and humus stability, which is of great significance for improving the soil quality of saline–alkali land and enhancing the productivity of fruit mulberry.

## Figures and Tables

**Table 1:** Biochemical characteristics of the isolated *Bacillus fexus* E1.

Oxidase	Hydrolyzed Starch	V-P Test	D-Glucose	D-Mannitol	Methyl Red	Utilization of Nitrate	Motility	Sucrose
+ ^a^	−	−	−	−	+	+	+	−

^a^ +: positive; −: negative.

**Table 2:** Physicochemical properties of the peanut shell biochar.

pH	EC (μS∙cm^−1^)	Total C (%)	Total N (g∙kg^−1^)	Total P (g∙kg^−1^)	Total K (g∙kg^−1^)	CEC (cmol∙kg^−1^)
8.98	1450	41.77	25.20	0.70	10.70	11.00

**Table 3:** Effects of different treatments on physical properties of the soil used for planting fruit mulberry seedlings.

Treatment	Bulk Density (g∙cm^−3^)	Field Water-Holding Capacity (g∙kg^−1^)	Total Porosity (%)	Capillary Porosity (%)	Non-Capillary Porosity (%)	Water Stable Macro-Aggregate (%)
CK	1.33 ± 0.02 b	195.33 ± 11.59 a	49.81 ± 0.65 b	25.99 ± 1.76 abc	23.82 ± 2.26 b	22.53 ± 0.67 b
SS	1.41 ± 0.04 a	209.00 ± 15.62 a	46.67 ± 1.57 c	29.51 ± 1.64 a	17.16 ± 1.66 c	20.73 ± 1.20 c
SS+P	1.38 ± 0.03 ab	199.67 ± 28.50 a	48.05 ± 1.21 bc	27.44 ± 3.43 ab	20.61 ± 2.75 bc	21.70 ± 1.22 bc
SS+B	1.34 ± 0.04 b	186.00 ± 3.61 a	49.43 ± 1.51 b	24.93 ± 1.00 bc	24.51 ± 2.44 b	25.33 ± 0.51 a
SS+P+B	1.27 ± 0.04 c	185.00 ± 6.56 a	52.20 ± 1.33 a	23.42 ± 0.18 c	28.78 ± 1.15 a	26.50 ± 0.66 a

Note: Data are mean ± SD. CK: control; SS: salt stress; SS+P: salt stress + PGPR; SS+B: salt stress + biochar; SS+P+B: salt stress + PGPR + biochar. Different letters indicate significant differences among treatments at *p* < 0.05 by LSD.

**Table 4:** Effects of different treatments on the basic chemical properties of fruit mulberry seedling rhizosphere soil.

Treatment	pH	EC (μS·cm^−1^)	Alkali-Hydrolyzed N (mg·kg^−1^)	Available P (mg·kg^−1^)	Available K (mg·kg^−1^)	SOM (g·kg^−1^)	CEC (cmol·kg^−1^)
CK	6.78 ± 0.03 c	378.67 ± 12.90 d	32.20 ± 2.10 a	10.84 ± 0.98 ab	80.33 ± 2.72 b	16.83 ± 1.08 b	12.27 ± 0.06 b
SS	6.73 ± 0.03 d	1558.67 ± 20.55 b	30.57 ± 1.46 a	12.24 ± 0.90 a	72.59 ± 0.63 c	16.17 ± 1.12 b	12.47 ± 0.59 ab
SS+P	6.78 ± 0.03 c	1499.33 ± 13.20 c	28.00 ± 2.10 a	10.65 ± 0.86 ab	62.07 ± 1.50 e	16.93 ± 0.45 b	12.80 ± 0.20 ab
SS+B	7.01 ± 0.01 b	1575.67 ± 30.24 b	31.50 ± 3.21 a	10.46 ± 0.96 b	89.19 ± 3.33 a	21.77 ± 0.60 a	12.47 ± 0.60 ab
SS+P+B	7.12 ± 0.01 a	1668.00 ± 12.29 a	28.00 ± 2.42 a	10.02 ± 0.50 b	66.64 ± 2.31 d	22.87 ± 0.40 a	13.30 ± 0.70 a

Note: Data are mean ± SD. CK: control; SS: salt stress; SS+P: salt stress + PGPR; SS+B: salt stress + biochar; SS+P+B: salt stress + PGPR + biochar; EC: electrical conductivity; SOM: soil organic matter; CEC: cation exchange capacity. Different letters indicate significant differences among treatments at *p* < 0.05 by LSD.

**Table 5:** Effects of different treatments on the enzyme activities in fruit mulberry seedling rhizosphere soil.

Treatment	Catalase (mg/g·h)	Urease (mg/g·24 h)	Sucrase (mg/g·24 h)	Neutral Phosphatase (mg/g·h)
CK	18.00 ± 0.70 b	0.52 ± 0.06 b	30.67 ± 3.98 a	0.02 ± 0.00 a
SS	18.23 ± 1.08 b	0.55 ± 0.03 b	29.53 ± 7.97 a	0.02 ± 0.01 a
SS+P	19.43 ± 0.47 ab	0.43 ± 0.06 b	40.63 ± 5.14 a	0.01 ± 0.01 a
SS+B	19.23 ± 2.06 ab	1.11 ± 0.21 a	42.20 ± 6.78 a	0.02 ± 0.01 a
SS+P+B	21.00 ± 0.56 a	1.43 ± 0.61 a	39.60 ± 7.70 a	0.02 ± 0.01 a

Note: Data are mean ± SD. CK: control; SS: salt stress; SS+P: salt stress + PGPR; SS+B: salt stress + biochar; SS+P+B: salt stress + PGPR + biochar. Different letters indicate significant differences among treatments at *p* < 0.05 by LSD.

## Data Availability

The data that support the findings of this study are available from the corresponding author upon reasonable request.

## Supplementary Materials

The following supporting information can be downloaded at https://www.mdpi.com/article/10.3390/biology14101441/s1, Supplementary File S1: The 16S RNA gene sequence of *B. fexus* E1 used in our study.
